# Sodium balance, circadian BP rhythm, heart rate variability, and intrarenal renin–angiotensin–aldosterone and dopaminergic systems in acute phase of ARB therapy

**DOI:** 10.14814/phy2.13309

**Published:** 2017-06-02

**Authors:** Yukako Isobe‐Sasaki, Michio Fukuda, Yoshiaki Ogiyama, Ryo Sato, Toshiyuki Miura, Daisuke Fuwa, Masashi Mizuno, Tetsuhei Matsuoka, Hiroko Shibata, Hiroyuki Ito, Minamo Ono, Sumiko Abe‐Dohmae, Ken Kiyono, Yoshiharu Yamamoto, Hiroyuki Kobori, Makoto Michikawa, Junichiro Hayano, Nobuyuki Ohte

**Affiliations:** ^1^ Department of Cardio‐Renal Medicine and HypertensionNagoya City University Graduate School of Medical SciencesNagoyaJapan; ^2^ Department of BiochemistryNagoya City University Graduate School of Medical SciencesNagoyaJapan; ^3^ Department of Mechanical Science and BioengineeringOsaka UniversityOsakaJapan; ^4^ Department of Physical and Health EducationUniversity of Tokyo Graduate School of EducationTokyoJapan; ^5^ International University of Health and WelfareTokyoJapan; ^6^ Department of Medical EducationNagoya City University Graduate School of Medical SciencesNagoyaJapan

**Keywords:** Angiotensinogen, dopamine, heart rate variability, chronic kidney disease

## Abstract

We have revealed that even in humans, activated intrarenal renin–angiotensin–aldosterone system (RAAS) enhances tubular sodium reabsorption to facilitate salt sensitivity and nondipper rhythm of blood pressure (BP), and that angiotensin receptor blocker (ARB) could increase daytime urinary sodium excretion rate (U_N__a_V) to produce lower sodium balance and restore nondipper rhythm. However, the sympathetic nervous system and intrarenal dopaminergic system can also contribute to renal sodium handling. A total of 20 patients with chronic kidney disease (61 ± 15 years) underwent 24‐h ambulatory BP monitoring before and during two‐day treatment with ARB, azilsartan. Urinary angiotensinogen excretion rate (U_AGT_V,* μ*g/gCre) was measured as intrarenal RAAS; urinary dopamine excretion rate (U_DA_V, pg/gCre) as intrarenal dopaminergic system; heart rate variabilities (HRV, calculated from 24‐h Holter‐ECG) of non‐Gaussianity index *λ*_25s_ as sympathetic nerve activity; and power of high‐frequency (HF) component or deceleration capacity (DC) as parasympathetic nerve activity. At baseline, glomerular filtration rate correlated inversely with U_AGT_V (*r* = −0.47, *P* = 0.04) and positively with U_DA_V (*r* = 0.58, *P* = 0.009). HF was a determinant of night/day BP ratio (*β *= −0.50, *F* = 5.8), rather than DC or *λ*_25s_. During the acute phase of ARB treatment, a lower steady sodium balance was not achieved. Increase in daytime U_N__a_V preceded restoration of BP rhythm, accompanied by decreased U_AGT_V (*r* = −0.88, *P* = 0.05) and increased U_DA_V (*r* = 0.87, *P* = 0.05), but with no changes in HRVs. Diminished sodium excretion can cause nondipper BP rhythm. This was attributable to intrarenal RAAS and dopaminergic system and impaired parasympathetic nerve activity. During the acute phase of ARB treatment, cooperative effects of ARB and intrarenal dopaminergic system exert natriuresis to restore circadian BP rhythm.

## Introduction

Increased salt sensitivity of blood pressure (BP) and nondipper type circadian BP rhythm are strongly associated with each other. Dahl et al., showed that salt sensitivity of BP is determined by the kidney (Dahl et al. 1962; Dahl and Heine 1975). Kimura and Brenner have revealed the nature of salt‐sensitive hypertension as impaired renal capability to excrete sodium into urine, which can originate from reduced glomerular ultrafiltration coefficient and/or from augmented tubular sodium (Na) reabsorption rate (t_Na_) (Kimura and Brenner 1995, 1997). In support of the former mechanism, we found inverse relationships between glomerular filtration rate (GFR) and night/day ratios of BP and urinary sodium excretion rate (U_Na_V) in patients with chronic kidney disease (CKD) (Fukuda et al. 2004, 2006). Patients with diminished renal sodium excretion (i.e., high‐salt sensitivity) can have sodium retention during the day, which prevents night‐time BP dip (i.e., nondipper circadian BP rhythm) (Fukuda et al. 2008a; Fukuda and Kimura 2012). In support of the latter mechanism, augmented t_Na_ caused by an inappropriately accelerated intrarenal renin–angiotensin–aldosterone system (RAAS) also impairs renal sodium excretion, eliciting the nondipper BP rhythm in patients with IgA nephropathy (Fukuda et al. 2012a). We also proved that treatment with an angiotensin (Ang) II type 1 receptor blocker (ARB), which can inhibit t_Na_, results in a lower sodium balance to restore nondipper circadian BP rhythm, accompanied by increased daytime U_Na_V, during the chronic phase (8 weeks) of treatment (Fukuda et al. 2008b, 2011, 2012b). Daytime U_Na_V is greater than night‐time U_Na_V in patients with preserved renal function, whereas daytime U_Na_V decreases and night‐time U_Na_V increases as renal capacity for sodium excretion is diminished (Koopman et al. 1989; Staessen et al. 1993; Centonza et al. 2000; Fukuda et al. 2004; Bankir et al. 2008). Thus, decreased daytime U_Na_V and increased night‐time U_Na_V are pathophysiologic conditions, and the increase in daytime U_Na_V and decrease in night‐time U_Na_V occur to normalize the circadian rhythm. Several basic studies have verified that Ang II enhances t_Na_, which can be inhibited by ARB, at various segments along the nephron (Barreto‐Chaves and Mello‐Aires 1996; Quan and Baum 1996; Peti‐Peterdi et al. 2002; Beutler et al. 2003). In patients with CKD, ARBs decrease the urinary potassium (K) excretion rate (U_K_V) to U_Na_V ratio, indicating suppression of function of the epithelial sodium channel (ENaC) (Ogiyama et al. 2014). Previously, we reported that an increase in daytime U_Na_V in CKD patients can precede restoration of nondipper circadian BP rhythm within 2 days after the start of ARB treatment (acute phase) (Miura et al. 2014).

The intrarenal dopaminergic system can also modify the renal capacity of natriuresis. Under salt deprivation, Ang II has the central role in t_Na_ (Crowley and Coffman 2007), whereas under a high‐salt diet dopamine (DA) secreted by renal proximal tubules acts as a paracrine substance to inhibit t_Na_ along the proximal to distal nephron (Bertorello et al. 1988; Bertorello and Aperia 1990; Olsen 1998; Carey 2001; Féraille and Doucet 2001; Gildea et al. 2012). DA excreted into urine is almost exclusively formed within the kidney (Lee 1993; Carey 2001). The sympathetic nervous system can also stimulate t_Na_, whereas ARBs inhibit central and peripheral sympathetic nerve activity (Ye et al. 2002). Recently, we proposed an increase in a non‐Gaussianity index of heart rate variability (HRV), *λ*_25s_, which indicates the probability of volcanic heart rate deviations that depart from each standard deviation level, as a marker of sympathetic cardiac overdrive (Kiyono et al. 2008; Hayano et al. 2011); and reported that L/T‐type calcium channel blocker, azelnidipine, which decreases sympathetic nerve activity (Shokoji et al. 2005; Konno et al. 2008; Inomata et al. 2014), reduced *λ*_25s_ in patients with CKD (Fukuda et al. 2016). However, relationships among circadian BP rhythm, U_Na_V, the intrarenal RAAS and dopaminergic system, and HRV during the acute phase of ARB treatment have yet to be clarified in patients with CKD.

### Glossary

**Table:** 

Sodium balance	
GFR	Glomerular filtration rate
S_Na_ × GFR	Amount of Na filtered from glomerulus and loaded into renal tubules (filtered tubular Na load, mmol/day): calculated as the product of the GFR and plasma Na concentration (S_Na_)
t_Na_	Tubular sodium reabsorption rate (mmol/day): calculated as the difference between filtered Na load and absolute urinary Na excretion (U_Na_V) t_Na_ = S_Na_ × GFR − U_Na_V
FR_Na_	Fractional tubular Na reabsorption (FR_Na_): calculated as the t_Na_ to filtered Na load ratio. FR_Na_ = t_Na_/(S_Na_ × GFR) = [S_Na_ × GFR − U_Na_V, mmol/day]/(S_Na_ × GFR, mmol/day) = 1 − U_Na_V/(S_Na_ × GFR)

Endocrine variables	
AGT	Angiotensinogen
Ang I	Angiotensin I
Ang II	Angiotensin II
AT1R	AngII type 1 receptor
AT2R	AngII type 2 receptor
ARB	Angiotensin II type 1 receptor blocker
hANP	Human atrial natriuretic peptide
U_AGT_V	Urinary angiotensinogen excretion rate
U_DA_V	Urinary dopamine excretion rate

Heart rate variability (HRV)	
* λ*_25s_	Non‐Gaussianity index *λ*_25s_: indicator of sympathetic nerve activities
DC	Deceleration capacity: indicator of parasympathetic nerve activities
HF	Power of high‐frequency component: indicator of parasympathetic nerve activities

## Material and Methods

### Subjects

A single‐arm, open label study was performed during hospitalization in 20 consecutive patients with CKD (14 men and six women; 61 ± 15 years; body mass index (BMI): 23.0 ± 3.8 kg/m^2^). Patients had to fulfill the following eligibility criteria: (1) age ≥16 years; (2) diagnosis of CKD based on Kidney Disease Outcomes Quality Initiative (K/DOQI) criteria (National Kidney Foundation, 2002) (GFR<60 mL/min/1.73 m^2^, or GFR ≥60 mL/min/1.73 m^2^ with accompanying proteinuria, defined as >300 mg per gram creatinine [/gCre]) for at least 3 months. GFR was estimated by Japanese equation for eGFR (Matsuo et al. 2009): GFR = 194 × [serum creatinine, mg/dL]^−1.094^ × [age, years]^−0.287^ × [one for male; 0.739 for female]; (3) decision of attending doctor to administer azilsartan (office BP >130/80 mmHg, or 125/75 mmHg if proteinuria ≥1 g/day on at least one occasion); (4) average sodium to creatinine ratio in spot urine measured at three different times for 4 weeks prior to enrollment <85–120 mmol/gCre (corresponding to ~6 g NaCl for daily intake based on expected daily urinary creatinine excretion as referred to hereinafter); and (5) written informed consent obtained. Patients were excluded if they had (1) treatment with ARBs or diuretics 2 months before enrollment; (2) change of antihypertensive agents in the 2 months before enrollment; (3) contraindication to azilsartan (history of allergic reactions to the drug, or renal artery stenosis); (4) the presence or possibility of pregnancy or current breastfeeding; (5) hemoglobin A1C (HbA1c) ≥9.0%; (6) GOT >100 or GPT >85; (7) endocrine hypertension; (8) accelerated or malignant hypertension (progressive renal dysfunction with diastolic BP (DBP) >120–130 mmHg; (9) serious conditions with congestive heart failure, coronary disease, arrhythmia, or systemic diseases; (10) nephrotic syndrome (serum albumin <2.5 g/dL); and (11) any reason for ineligibility suggested by the attending doctor (e.g., insufficient capacity to understand study content). All subjects were enrolled after providing informed consent to participation in the study. The study protocol was approved by the Institutional Review Board (IRB) of Nagoya City University Hospital (IRB approval number: 45‐12‐0022, UMIN registration number: 000009549) and was conducted in accordance with the Declaration of Helsinki, and the helm of the Ministry of Health, Labour and Welfare.

At the time of enrollment, 11 patients were taking no antihypertensive agents and nine were taking calcium channel blockers. Office BP was measured with a validated automated oscillometric sphygmomanometer (MPV3301, Nihon Koden, Tokyo, Japan) after subjects had been seated for at least 5 min. Office systolic and diastolic BP (SBP and DBP) determined as averages of two BP readings in two visits were 150 ± 16 and 87 ± 14 mmHg, respectively, and the spot‐urine sodium to creatinine ratio was 98 ± 18 mmol/gCre.

### Study protocol

The study was performed to evaluate relationships among circadian BP rhythm, U_Na_V, and intrarenal RAAS and dopaminergic system and HRV during the acute phase of ARB treatment in patients with CKD under hospitalization, during which patients ate a diet containing 6 g/day of salt. The patients received nutritional instructions to eat this diet for at least 4 weeks before enrollment, and were asked to get up at 06:00 and to start bed rest at 21:00 during the study period. Measurements were made before initiation of ARB treatment (baseline) and 2 days after the start of oral administration of an ARB, azilsartan (acute phase). After baseline examinations, subjects received single daily doses of azilsartan once in the morning. The daily dose was determined based on the average of two office SBP readings in two visits: ≥160 mmHg, 40 mg; ≥125 and <160 mmHg, 20 mg; and <125 mmHg, 10 mg. The number of patients taking azilsartan 40, 20, and 10 mg/day were 2, 12, and 6, respectively. During the two‐day study period, a change in dosage of azilsartan or other antihypertensive agents and additional administration of antihypertensives or diuretics were not allowed; if these were needed, the patient was excluded from the study. When SBP, which was measured thrice a day on the arm opposite to that used for 24‐h ambulatory BP monitoring (ABPM), fell below 100 or 95 mmHg, or the patient felt postural dizziness, the dose of the antihypertensive agent was decreased and the study was discontinued for the patient.

At baseline and in the acute phase of ARB treatment, 24‐h ABPM and urine collection were conducted separately in the daytime (06:00–21:00) and night‐time (21:00– 06:00), and ambulatory 24‐h ECG was recorded on the same day during normal daily activities. Collected urine was combined to calculate 24 h values. The adequacy of 24‐h urine collection was judged by the amount of urinary creatinine excretion: males aged <50 years, 18.5–25.0; females aged <50 years, 16.5–22.4; males aged ≥50 years, 15.7–20.2; and females aged ≥50 years, 11.8–16.1 mg/kg body weight per day. Incomplete or excessive urine collection in daytime or night‐time samples was judged based on the night/day urinary creatinine excretion ratio of <0.5 or >2.0, respectively. 24‐h creatinine clearance (ml/min) was used as an index for GFR. The glomerulotubular Na balance was defined as follows: the amount of Na filtered from glomerulus and loaded into renal tubules (filtered tubular Na load) was calculated as the product of the GFR and plasma Na concentration (S_Na_), S_Na_ × GFR. Tubular Na reabsorption (t_Na_) was calculated as the difference between filtered Na load and absolute urinary Na excretion (Koopman et al. 1989; Crowley and Coffman 2007). Fractional tubular Na reabsorption (FR_Na_) was then calculated as the t_Na_ to filtered Na load ratio. “Steady state treatment with an ARB” was defined as 24‐h U_Na_V returning to the same as the baseline level, and “lower Na balance on steady state” as (1) filtered tubular Na load reduced, (2) tubular Na reabsorption reduced, and (3) 24‐h U_Na_V returning to the same as the baseline level.

Blood samples at baseline and in the acute treatment phase were collected at 06:00, the boundary between day and night. Plasma Na and K (ion‐selective electrode method) and creatinine (enzymatic method), and urinary concentrations of Na and K (ion‐selective electrode method), creatinine (enzymatic method), and albumin (turbidimetric immunoassay) were measured at the institutional central laboratory. Blood samples for evaluating plasma renin activity (PRA), plasma concentrations of aldosterone (PAC), Ang I, Ang II, adrenaline (AD), noradrenaline (NAD), dopamine (DA), and human atrial natriuretic peptide (hANP), and urine samples for AD, NAD, and DA were centrifuged at 1500 *g* for 10 min at 4°C, frozen immediately, and stored at −35°C until assay. Assays were performed for PRA, PAC, Ang I, and Ang II by radioimmunoassay; serum and urinary AD, NA and DA by high‐performance liquid chromatography (HPLC); and hANP by chemiluminescent enzyme immunoassay (CLEIA) at an external center (SRL Inc., Hachioji, Japan). Plasma AD and DA concentrations fell below the lower limit of detection in six and nine patients, respectively. For these patients, the data were taken to be 50% of the detection limit (i.e., 2.5 pg/mL for AD and DA). Urinary angiotensinogen (AGT) was measured using a Human Total AGT ELISA Kit (Immuno‐Biological Laboratories Co. Takasaki, Japan) (Katsurada et al. 2007), with intra‐ and interassay coefficients of 4.4% and 4.3%, respectively (Suzaki et al. 2006; Katsurada et al. 2007). Urinary AGT excretion (U_AGT_V, *μ*g/gCre) and urinary DA excretion (U_DA_V, pg/gCre) were used as indicators of activities of the intrarenal RAAS and dopaminergic system, respectively. For reference, urinary excretion rates of AD and NAD (U_AD_V and U_NAD_V, pg/gCre) were calculated.

### 24‐h ABPM analysis

During 24‐h BP monitoring, BP was monitored noninvasively every 30 min with a validated automatic device (model TM‐2425; A&D, Tokyo, Japan). BP and heart rate (HR) values were not considered valid for analysis if data were missing continuously for 2 h or if the patient awoke during the night and had difficulty falling asleep again. Mean arterial pressure (MAP) was calculated as DBP plus one‐third of the pulse BP. Daytime BP was calculated as the average of the 30 readings between 06:00 and 21:00, and night‐time BP as the average of the remaining 18 readings. Patients whose nocturnal fall in MAP was ≥10% from day to night were classified as dippers and those with a nocturnal MAP fall <10% as nondippers. Nocturnal hypertension was defined as night‐time BP ≥120/70 mmHg.

### HRV analysis

Twenty‐four‐hour ECG was recorded with a portable recorder (RAC‐3103, Nihon Koden, Tokyo, Japan). Ambulatory ECG signals were digitized at 125 Hz and 12 bits with an ECG scanner (DSC‐3300, Nihon Koden), on which QRS complexes were detected and labeled automatically and all possible errors in labeling were reviewed and edited manually by experienced technicians. Recordings with a total analyzable length <23.5 h were excluded from the study. Data were also excluded when ventricular and supraventricular ectopic beats were >10% of all recorded beats. Only normal‐to‐normal R‐R interval data thus obtained were used for HRV analysis. Among HRVs, we used *λ*_25s_ as an indicator of sympathetic nerve activity, and power of high frequency (HF, 0.15–0.40 Hz) and deceleration capacity (DC) for parasympathetic nerve activity. We hypothesized that DC could be attributable to both sympathetic and parasympathetic nerve activities (Fukuda et al. 2016). These HRVs were calculated as described previously (Fukuda et al. 2016). In brief, *λ*_25s_ was calculated to characterize the non‐Gaussian nature of HRV and to detect intermittency of the HR increment (Kiyono et al. 2008; Hayano et al. 2011). This index was derived from a method for analysis of multiscale statistics of complex fluctuations, and originally used for characterizing intermittency of hydrodynamic turbulence. *λ*_25s_ indicates probabilities of a volcanic HR deviation of departure from each SD levels, and a larger value of *λ*_25s_ indicates that the observed distribution of HRV has fatter tails and a sharper peak compared with a normal Gaussian distribution, which displays no broad base or fat tails (Fukuda et al. 2016). Recently, we proposed *λ*_25s_ as a marker of sympathetic cardiac overdrive (Kiyono et al. 2008; Hayano et al. 2011), and showed that an increase in *λ*_25s_ is associated exclusively with increased cardiac mortality risk independent of clinical risk factors and other HRVs in patients with a history of acute myocardial infarction (Hayano et al. 2011). We also reported that L/T‐type calcium channel blocker, azelnidipine, which decreases sympathetic nerve activity (Shokoji et al. 2005; Konno et al. 2008; Inomata et al. 2014), reduced *λ*_25s_ in patients with CKD (Fukuda et al. 2016). DC was measured by Bauer's signal processing technique of phase‐rectified signal averaging. The technique gives separate characterizations of deceleration‐ and acceleration‐related modulations to distinguish conceptually between vagal and sympathetic factors affecting HRV, and quantifies them as DC and acceleration capacity, respectively (Bauer et al. 2006).

Control data for the HRV analysis were obtained from age‐, gender‐, and BMI‐matched same number of persons, including individuals who underwent 24‐h ambulatory ECG for evaluation of chest discomfort without medication with antihypertensive agents, but were proven not to have cardiac and kidney diseases or hypertension, or healthy volunteers (*n* = 20). For ethical reasons, the ARB was not started in these 20 persons.

### Statistical analysis

Results are expressed as mean ± SD or as median (interquartile range, IQR) according to the data distribution, which was tested using a Kolmogorov–Smirnov test. Variables that were not normally distributed were analyzed after log transformation. Differences in parameters between control and baseline, or baseline and ARB treatment were examined by Student's t‐test for paired samples or by Wilcoxon signed‐rank test, as appropriate. Correlations among quantitative variables were evaluated by the least‐squares method. Relationships between changes in variables were analyzed by linear regression through the origin. Considering the statistical power of our study, which included 20 patients, we could use only 2–3 covariates for multiple regression analysis. Therefore, we used forward stepwise multiple regression analysis only when we evaluated the important clinical questions. In particular, forward stepwise multiple regression analysis was conducted to compare the contribution of *λ*_25s_, DC, and HF to nocturnal BP or night/day BP ratio, and the contribution of changes in U_AGT_V, U_DA_V, and *λ*_25s_ to the change in filtered tubular Na load. A value of *P *<* *0.05 was considered to be significant. Statistical analyses were performed using SPSS Statistics 22 (IBM Corp., NY).

## Results

### Baseline

At baseline, the median (IQR) for albuminuria was 370 (67–870) mg/gCre and the mean±SD GFR was 60 ± 42 mL/min/1.73 m^2^. BP, HR, and urinary excretion of sodium and potassium are shown in Table 1. All 20 patients had nocturnal hypertension. Five of the 20 patients exhibited a dipper type circadian BP rhythm and 15 had a nondipper rhythm. GFR correlated inversely with 24‐h (*r* = −0.43, *P* = 0.05), daytime (*r* = −0.34, *P* = 0.1), and night‐time (*r* = −0.53, *P* = 0.02) SBP. The relationship between GFR and FR_Na_ exhibited an upward convex relationship (*r* ^2^ = 0.66, *P* = 0.0001), rather than a first‐order regression line (*r* ^2^ = 0.32, *P* = 0.009).

**Table 1:** Blood pressure, heart rate, and urinary excretion of sodium and potassium before and during the acute phase of ARB treatment

Variable		Baseline	ARB	*P*‐value
SBP	24 h (mmHg)	142 ± 19	132 ± 18	0.0001
	Day (mmHg)	143 ± 19	135 ± 20	0.001
	Night (mmHg)	139 ± 20	126 ± 23	0.0007
	Night/day ratio	0.97 ± 0.09	0.93 ± 0.12	0.04
DBP	24 h (mmHg)	84 ± 12	78 ± 10	0.004
	Day (mmHg)	86 ± 12	81 ± 11	0.01
	Night (mmHg)	80 ± 12	71 ± 9	0.003
	Night/day ratio	0.94 ± 0.06	0.88 ± 0.11	0.02
MAP	24 h (mmHg)	103 ± 12	96 ± 11	0.001
	Day (mmHg)	105 ± 12	99 ± 12	0.003
	Night (mmHg)	100 ± 12	89 ± 12	0.0007
	Night/day ratio	0.96 ± 0.07	0.91 ± 0.11	0.02
HR	24 h (rpm)	70 ± 10	72 ± 9	0.3
	Day (rpm)	73 ± 10	75 ± 9	0.04
	Night (rpm)	65 ± 10	66 ± 11	0.09
	Night/day ratio	0.89 ± 0.08	0.88 ± 0.08	0.2
U_Na_V	24 h (mmol/gCre)	86 ± 46	70 ± 34	0.04
	Day (mmol/h)	3.58 ± 2.44	3.02 ± 1.68	0.2
	Night (mmol/h)	3.60 ± 2.04	2.75 ± 1.37	0.02
	Night/day ratio	1.23 ± 0.77	1.10 ± 0.77	0.2
U_K_V	24 h (mmol/gCre)	20 ± 10	18 ± 11	0.3
	Day (mmol/h)	0.87 ± 0.53	0.82 ± 0.60	0.7
	Night (mmol/h)	0.77 ± 0.38	0.61 ± 0.31	0.03
	Night/day ratio	0.96 ± 0.35	0.82 ± 0.30	0.1
U_K_V/U_Na_V	24 h	0.27 ± 0.16	0.27 ± 0.10	0.8
	Day	0.29 ± 0.18	0.29 ± 0.14	0.9
	Night	0.26 ± 0.17	0.25 ± 0.13	0.6
	Night/day ratio	0.90 ± 0.28	0.91 ± 0.42	0.9

Values are shown as mean ± standard deviation (*n = 20*). ARB, angiotensin receptor blocker; SBP, systolic blood pressure; DBP, diastolic blood pressure; MAP, mean arterial pressure; HR, heart rate; U_Na_V, U_K_V and U_Na_V/U_K_V; urinary excretion rates of sodium, potassium; and U_Na_V to U_K_V ratio, respectively.

**Figure 1:**
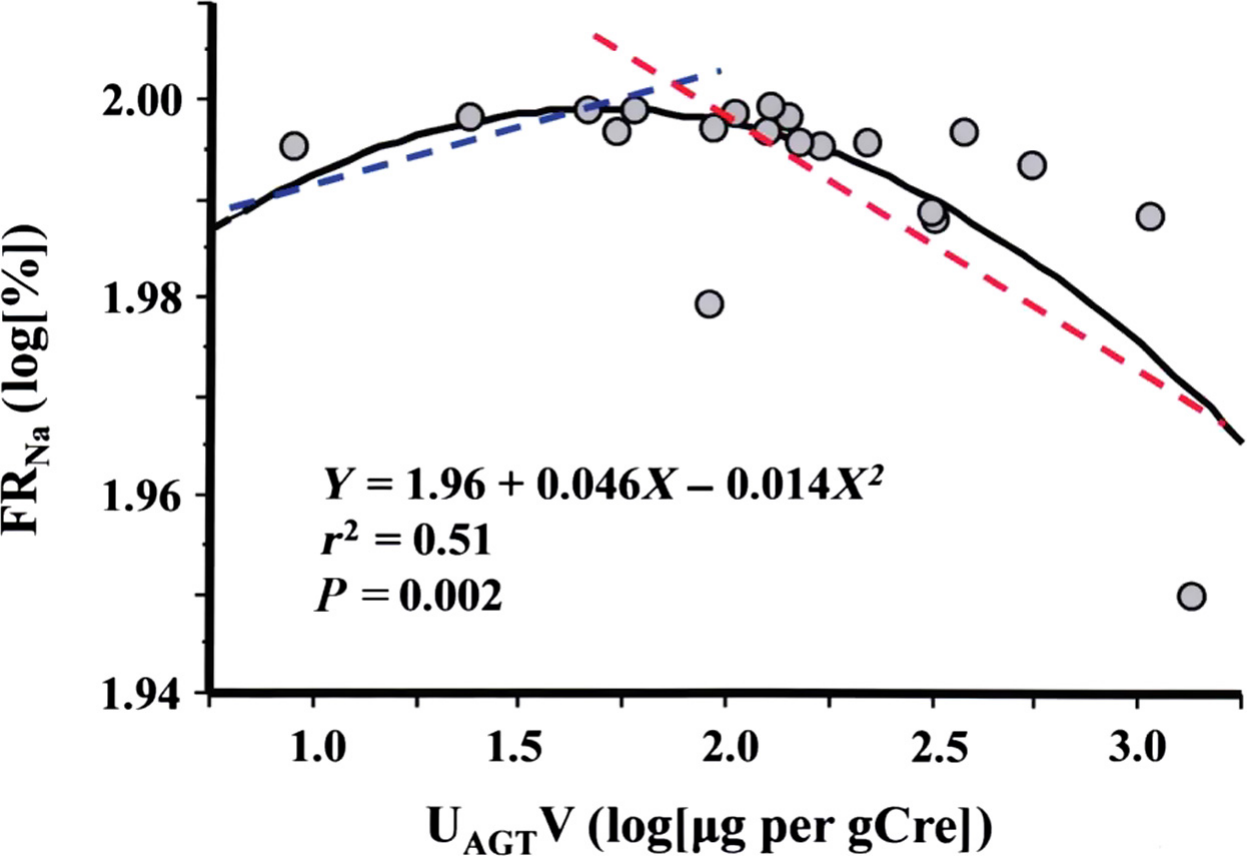
Convex relationship between urinary excretion rate of angiotensinogen (U_AGT_V) and fractional tubular sodium reabsorption (FR_N__a_) at baseline. U_AGT_V (*x*‐axis) and FR_N__a_ (*y*‐axis) exhibited an upward convex relationship. The peak of the curve was at a coordinate (*x* = 1.643, and *y* = 1.997) corresponding approximately to U_AGT_V of 43.9 *μ*g per gCre, and FR_N__a_ of 99.49%. In patients with lower U_AGT_V, U_AGT_V had a positive relationship with FR_N__a_ (blue line), whereas in patients with higher U_AGT_V, U_AGT_V had a negative relationship with FR_N__a_ (red line).

**Figure 2:**
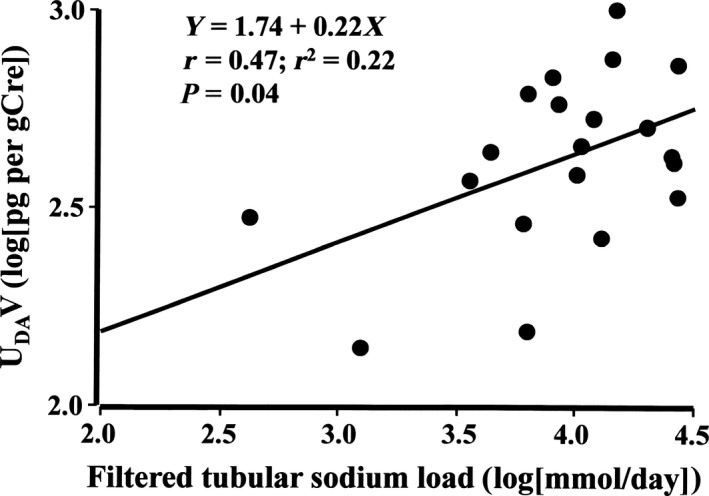
Urinary dopamine excretion rate (U_DA_V) exhibited a direct correlation with 24‐h filtered tubular sodium load. This relationship was consistent with findings from basic studies showing that as the amount of sodium delivered to proximal tubules increases, dopamine secretion by the tubules is augmented.

Endocrine variables at baseline are shown in Table 2. Of note, plasma AD and DA concentrations fell below the lower limit of detection in 6 and 9 patients, respectively. However, although insignificant, U_DA_V was higher in patients whose plasma DA fell below the detection limit compared to those with a detectable plasma DA concentration (543 ± 268 vs. 393 ± 136 pg per gCre, *P* = 0.1), whereas U_AD_V was 6.9 ± 5.6 and 6.7 ± 4.0 pg/gCre in patients whose plasma AD could and could not be determined, respectively (*P* = 0.9). Night/day SBP ratio correlated inversely with PRA (*r* = −0.49, *P* = 0.03) and PAC (*r* = −0.49, *P* = 0.03), but not with other endocrine variables (Table 4). GFR correlated positively with U_DA_V (*r* = 0.58, *P* = 0.009) and inversely with U_AGT_V (*r* = −0.47, *P* = 0.04) and hANP (*r* = −0.52, *P* = 0.02), but not with others (Table 4). Urinary excretion of AGT (U_AGT_V) [log(*μ*g/gCre)] did not correlate with 24‐h, daytime, night‐time, and night/day ratios of SBP. U_AGT_V correlated positively with 24‐h U_Na_V (*r* = 0.60, *P* = 0.05) and hANP (*r* = 0.58, *P* = 0.007), but not with other endocrine variables or urinary albumin excretion rate (U_Alb_V). U_AGT_V and FR_Na_ exhibited an upward convex relationship (*r* ^2^ = 0.51, *P* = 0.002), rather than a first‐order regression line (*r* ^2^ = 0.28, *P* = 0.02, Fig. 1): log[FR_Na_] = 1.96 + 0.046 ×  log[U_AGT_V] ‐ 0.014 ×  [log(U_AGT_V)]^2^. The peak coordinate of the upward convex curve corresponded approximately to U_AGT_V of 43.9 *μ*g/gCre, and FR_Na_ of 99.49%. U_DA_V [log(pg/gCre)] correlated positively with 24‐h filtered Na load (*r* = 0.47, *P* = 0.04, Fig. 2), but not with endocrine variables or 24‐h U_K_V/U_Na_V ratio.

**Table 2:** Endocrine variables before and during ARB treatment

Variable	Baseline	ARB	*P*‐value
hANP (ng/mL/h)	40 ± 3	30 ± 3	0.001
PRA (ng/ml/h)	0.7 (0.5–1.0)	0.9 (0.6–2.7)	0.002
PAC (pg/mL)	95 ± 72	66 ± 45	0.002
Ang I (pg/mL)	95 (58–130)	125 (35–180)	0.2
Ang II (pg/mL)	7 (4–13)	10 (8–14)	0.2
U_AGT_V (*μ*g per gCre)	134 (82–315)	119 (57–161)	0.1
AD (pg/mL)	19 (3–39)	16 (12–30)	0.2
NAD (pg/mL)	232 (158–308)	271 (159–397)	0.1
DA (pg/mL)	8 (3–12)	12 (3–14)	0.4
U_AD_V (pg per gCre)	6.8 ± 4.7	9.3 ± 13.2	0.3
U_NAD_V (pg per gCre)	105.7 ± 49.6	128.6 ± 88.5	0.1
U_DA_V (pg per gCre)	464.2 ± 217.2	503.5 ± 254.2	0.5

Values are shown as mean ± standard deviation or median (interquartile range) (*n = 20*). ARB, angiotensin receptor blocker; hANP, human atrial natriuretic peptide; PRA, plasma renin activity; PAC, plasma aldosterone concentration; Ang I, angiotensin I; Ang II, angiotensin II; U_AGT_V; urinary excretion rate of angiotensinogen; AD, NAD and DA, plasma concentrations of adrenaline, noradrenaline and dopamine; U_AD_V, U_NAD_V and U_DA_V; urinary excretion rates of adrenaline, noradrenaline and dopamine.

HRVs at baseline are shown in Table 3. Baseline *λ*_25s_ was higher and DC was lower compared to control values. GFR correlated positively with DC (*r* = 0.45, *P* = 0.04), but not with HF or *λ*_25s_. 24‐h *λ*_25s_ did not show a significant correlation with 24‐h, daytime, night‐time, and night/day ratios of SBP. 24‐h DC correlated inversely with night‐time SBP (*r* = −0.45, *P* = 0.05), but not with other SBP variables. 24‐h HF correlated inversely with night‐time SBP (*r* = −0.49, *P* = 0.03) and night/day SBP ratio (*r* = −0.44, *P* = 0.05). 24‐h *λ*_25s_, DC, and HF did not have a significant relationship with endocrine variables (Table 4). In stepwise multiple regression analysis (*R* ^2^ = 0.19, *P* = 0.05), HF was the main determinant of night/day SBP ratio (*β *= −0.44, *F* = 4.3), rather than DC or *λ*_25s_.

**Table 3:** Changes in heart rate variabilities in the acute phase of ARB treatment

Variable	Control	Baseline	ARB	*P*‐value^a^
Frequency domain measures				
HF [ln(ms^2^)]	4.53 ± 1.41	4.82 ± 1.09	4.69 ± 1.24	0.3
Nonlinear measures				
DC (ms)	5.34 ± 2.18	6.73 ± 2.47^b^	6.60 ± 2.14	0.5
* λ*_25s_	0.41 ± 0.08	0.51 ± 0.15^b^	0.51 ± 0.14	0.8

Values are expressed as the mean ± SD (*n* = 20). ARB, angiotensin receptor blocker; Abbreviations for HRV measures are explained in the text.

a *P*‐values for baseline vs. ARB treatment.

b Difference in HRV between control and baseline was significant (*P* < 0.05).

**Table 4:** Correlations among endocrine variables and explanatory variables using simple linear regression analysis

Explanatory variables	Endocrine variables (objective variables)				
	PRA	PAC	hANP	U_AGT_V	U_DA_V
GFR	NS	NS	−0.52 0.02	−0.47 0.04	0.58 0.009
Night/day SBP	−0.49 0.03	−0.49 0.03	NS	NS	NS
U_AGT_V	NS	NS	0.58 0.007	―	NS
U_DA_V	NS	NS	NS	NS	―
*λ*_25s_	NS	NS	NS	NS	NS
DC	NS	NS	NS	NS	NS
HF	NS	NS	NS	NS	NS

In each cells, the number described above is correlation coefficients, and the number below is *P*‐values of simple linear regression analysis. NS, not significant; PRA, plasma renin activity; PAC, plasma aldosterone concentration; hANP, human atrial natriuretic peptide; Ang I, angiotensin I; Ang II, angiotensin II; U_AGT_V; urinary excretion rate of angiotensinogen; U_DA_V, urinary excretion rates of dopamine; GFR, glomerular filtration rate; *λ*_25s_, non‐Gaussianity index; DC, deceleration capacity; HF, power of high‐frequency component.

### Effects of add‐on HCTZ to ARB therapy

During the acute phase of azilsartan treatment, body weight (59.3 ± 14.4 to 59.2 ± 14.2 kg, *P* = 0.2), serum sodium (142 ± 3 to 141 ± 3 mEq/L, *P* = 0.3), and serum potassium (3.8 ± 0.5 to 3.8 ± 0.5 mEq/L, *P* = 0.9) remain unchanged, and serum creatinine increased (1.98 ± 1.94 to 2.04 ± 1.94 mg/dL, *P* = 0.04). Albuminuria changed from 370 (IQR, 67–870) to 270 (IQR, 68–1030) mg/gCre (*P* = 0.2), and GFR from 60 ± 42 to 57 ± 44 mL/min (*P* = 0.4). Clinical variables before and during treatment are shown in Tables 1, 2 and 3. Daytime, night‐time, 24‐h, and night/day ratios of SBP, DBP, and MAP were all lowered (Table 1). Circadian BP rhythm and night‐time BP profiles changed as follows. All five patients with dipper BP rhythm at baseline remained dipper, with three with persistent nocturnal hypertension and two reverting to nocturnal normotension. Of the 15 patients with a nondipper BP rhythm and nocturnal hypertension at baseline, 10 still had a nondipper BP rhythm (eight had persistent nocturnal hypertension and two reverted to nocturnal normotension) and five changed to dipper (three had persistent nocturnal hypertension and two reverted to nocturnal normotension). One of the two patients who changed to a dipper and nocturnal normotension had excess BP reduction during the acute phase of azilsartan therapy. hANP and PAC decreased, and PRA increased (Table 2). There was no significant difference in *λ*_25s_, DC, and HF between baseline and the acute phase of treatment (Table 3).

Relationships between change in SBP variables and study measurements are shown in Table 5. Change in PRA exhibited significant inverse correlations with changes in absolute values of 24‐h, daytime and night‐time SBP, whereas change in PAC correlated positively with night‐time SBP. Of note, the change in body weight showed no significant correlation with changes in absolute SBP values and in night/day SBP ratio.

**Table 5:** Correlations among blood pressure variables and explanatory variables using simple linear regression analysis

	Changes in Systolic BP			
	24 h	Day	night	night/day
PRA	−0.62 0.003	−0.64 0.002	−0.51 0.01	NS
PAC	NS	NS	0.43 0.05	NS
hANP	NS	NS	NS	NS
U_AGT_V	NS	NS	NS	NS
U_DA_V	NS	NS	NS	NS
*λ*_25s_	NS	NS	NS	NS
DC	NS	NS	NS	NS
HF	NS	NS	NS	NS

In each cells, the number described above is correlation coefficients, and the number below is *P*‐values of simple linear regression analysis. NS, not significant; PRA, plasma renin activity; PAC, plasma aldosterone concentration; hANP, human atrial natriuretic peptide; U_AGT_V; urinary excretion rate of angiotensinogen; U_DA_V, urinary excretion rates of dopamine; *λ*_25s_, non‐Gaussianity index; DC, deceleration capacity; HF, power of high‐frequency component.

Change in filtered Na load did not correlate with changes in hANP, U_DA_V, and HRVs (*λ*_25s_, DC, and HF). Change in daytime U_Na_V correlated inversely with change in PRA (*r* = −0.51, *P* = 0.02), but not with changes in other endocrine or HRV variables. Changes in 24‐h U_K_V/U_Na_V ratio correlated inversely with change in U_DA_V (*r* = −0.44, *P* = 0.05), but not with changes in other variables. Change in U_AGT_V did not correlate with changes in endocrine or HRV variables.

As mentioned above, of the 15 patients with nondipper BP rhythm and nocturnal hypertension at baseline, five changed to dipper. One of these five patients had excess BP reduction during the acute phase of azilsartan therapy and his restoration of nondipper BP rhythm was not accompanied by an increase in daytime U_Na_V. However, the other four patients with restoration of nondipper BP rhythm had an increase in daytime U_Na_V, consistent with our previous reports (Fukuda et al. 2008b, 2011, 2012b). In these four patients, change in daytime U_Na_V correlated inversely with change in U_AGT_V (*r* = −0.88, *P* = 0.05) and positively with change in U_DA_V (*r* = 0.87, *P* = 0.05), but did not correlate with changes in hANP, *λ*_25s_, HF, and DC. Change in U_DA_V was positively correlated with change in filtered tubular Na load (*r* = 0.96, *P* = 0.01). In stepwise multiple regression analysis (*R* ^2^ = 0.89, *P* = 0.05), change in U_DA_V was the main determinant of change in filtered tubular Na load (*β *= 0.94, *F* = 16.3), rather than changes in U_AGT_V and *λ*_25s_.

The changes in glomerulotubular balances of sodium before and during the acute phase of azilsartan therapy were tubular sodium load 12202 ± 8624 to 11677 ± 9013 (*P* = 0.4), tubular sodium reabsorption 12116 ± 8619 to 11607 ± 9002 (*P* = 0.4), and urinary sodium excretion, 86 ± 46 to 70 ± 34 (*P* = 0.04).

## Discussion

### Sodium balance

The glomerulotubular balances of sodium before and during the acute phase of azilsartan therapy in this study indicated that a lower steady sodium balance had not been achieved. At baseline, an inverse relationship of GFR and hANP was found, supporting our hypothesis that as renal function deteriorated, body fluid retention occurred to cause nondipper circadian BP rhythm. However, reduction in nocturnal BP during ARB treatment was associated with increased PRA, but not with a change in hANP. This finding and the absence of a mechanism for the kidney to sense total body water volume suggest that the kidney itself, rather than total body water, determines salt sensitivity and circadian rhythm of BP. In fact change in body weight showed no significant correlation with changes in absolute BP values and in night/day BP ratio in our study. This reminds us of the work of Dahl, who established two strains of rats by selective inbreeding: (1) a salt‐sensitive rat (S) that becomes hypertensive under a high‐salt diet, and (2) a salt‐resistant rat (R) that remains normotensive under the same condition. Dahl also found that renal homograft from the R‐ to S‐rat made the rat salt‐resistive, whereas renal homograft from the S to R made the rat salt sensitive (Dahl et al. 1962; Dahl and Heine 1975). These findings indicate that salt sensitivity of BP is determined by the kidney.

Patients with diminished renal sodium excretion (i.e., high‐salt sensitivity) can incur sodium retention during the day, which prevents night‐time BP dip (i.e., nondipper circadian BP rhythm) (Fukuda and Kimura 2012). The duration from the time when subjects went to bed until the time when nocturnal BP dip first occurred (defined as dipping time, DT) is prolonged to exert pressure‐natriuresis until sufficient sodium is eliminated in patients with more severe renal dysfunction (Fukuda et al. 2008a). We also proved that treatment with an ARB, which can inhibit t_Na_, could result in a lower sodium balance to restore nondipper circadian BP rhythm accompanied by both increased daytime U_Na_V and shortened DT in the chronic phase (8 weeks) of treatment (Fukuda et al. 2008b, 2011, 2012b). A number of basic studies have verified that Ang II enhances t_Na_, which is inhibited by ARBs, at various segments along the nephron (Barreto‐Chaves and Mello‐Aires 1996; Quan and Baum 1996). Even when t_Na_ in the upper tubules is inhibited by ARBs, downstream t_Na_ can be enhanced. However, ARBs can also decrease the number (Beutler et al. 2003) and activity (Peti‐Peterdi et al. 2002) of ENaC, independent of circulating aldosterone, and can decrease the U_K_V/U_Na_V ratio, indicating suppression of ENaC function (Ogiyama et al. 2014). In this way, ARBs can enhance daytime U_Na_V, similar to that achieved with diuretics (Fukuda et al. 2008b, 2011), to restore nondipper circadian BP rhythm.

We have emphasized a close relationship between increased daytime U_Na_V and restoration of nondipper circadian BP rhythm (Fukuda et al. 2008b, 2011, 2012b). Recently, we examined whether an increase in daytime U_Na_V or a decrease in night‐time BP occurs first within 2 days after the start (acute phase) of ARB treatment in CKD patients (Miura et al. 2014). An increase in daytime U_Na_V is not attributable to BP reduction during the previous night. Rather, the increase in daytime U_Na_V precedes restoration of nondipper circadian BP rhythm. In fact, the present study showed that an increase in daytime U_Na_V preceded restoration of circadian BP rhythm in patients who were nondipper type at baseline. In these patients, change in daytime U_Na_V correlated inversely with change in U_AGT_V and positively with change in U_DA_V. These findings reflect studies showing that renal sodium excretion capability is attributable to both the intrarenal AngII (antinatriuretic) and dopaminergic (natriuretic) systems. Thus, AngII inhibits the natriuretic effect of dopamine (Choi et al. 2009). The current study is the first to investigate the relationship between U_AGT_V and U_DA_V as indicators of intrarenal RAAS and dopaminergic system activity, respectively, in patients with CKD.

### Intrarenal renin–angiotensin–aldosterone system

At baseline, U_AGT_V had a positive relationship with 24‐h U_Na_V. The finding is consistent with a previous report that salt intake enhances and salt‐restriction decreases intrarenal RAAS activity (Konishi et al. 2011). Overhydration essentially suppresses systemic RAAS, whereas overhydration accompanied by high‐salt intake accelerates intrarenal RAAS. In this way, intrarenal RAAS activity cannot be pictured from systemic RAAS activity. For instance, diabetes mellitus is strongly associated with low PRA and low PAC, but intrarenal RAAS is generally activated (Anderson et al. 1993; Burns and Harris 1995). We speculate that this is why, in the present study, U_AGT_V correlated positively with hANP, which increased as renal function deteriorated, rather than PRA. As renal function deteriorated, U_AGT_V increased, and U_AGT_V was not associated with U_Alb_V. These findings also provide a coherent explanation of why renal dysfunction enhances intrarenal RAAS activity, and suggest that the genesis of urinary AGT is not the same as that of urinary albumin, which is filtered through glomerular capillary walls.

Previously, we reported that proximal tubular expression of AGT, indicating intrarenal RAAS activity, showed a direct relationship with tubular sodium reabsorption (Fukuda et al. 2012a). However, in the present study U_AGT_V and FR_Na_ had an upward convex relationship (Fig. 1), rather than a first‐order regression line. Therefore, we have to consider the consistency of our present finding with the previous results regarding the relationship between U_AGT_V and FR_Na_. FR_Na_ can be calculated as follows: FR_Na_ = t_Na_/(S_Na_ × GFR) = [S_Na_ × GFR ‐ U_Na_V, mmol/day]/(S_Na_ × GFR, mmol/day) = 1 − U_Na_V/(S_Na_ × GFR). Thus, FR_Na_ is corresponding to U_Na_V adjusted by S_Na_ and GFR, indicating that relationship between U_AGT_V and FR_Na_ is controlled by GFR. U_Na_V and [S_Na_ × GFR] ranged from 50 to 110, and 8300 to 21,000 mmol/day, respectively; therefore, [S_Na_ × GFR] was several orders of magnitude higher than U_Na_V. U_AGT_V had an inverse correlation with GFR, and thus, higher values of U_AGT_V represent reduced GFR. Furthermore, GFR and FR_Na_ also had an upward convex relationship, and this may be why U_AGT_V showed an upward convex relationship with FR_Na_ in this study. Consequently, in patients with preserved GFR, FR_Na_ may have a positive relationship with U_AGT_V (blue line, Fig. 1), whereas in patients with deteriorated GFR, FR_Na_ has an inverse relationship with U_AGT_V (red line, Fig. 1). Renal function in the previous study population was homogenously preserved, and this may have lessened the impact of GFR on the results to produce a linear relationship of U_AGT_V and FR_Na_ (Fukuda et al. 2012a).

### Intrarenal dopaminergic system

Our study demonstrated that the plasma DA concentration fell below the lower limit of detection in nine of 20 patients, but U_DA_V could be measured in all 20 patients. U_DA_V was higher in these nine patients compared to the 11 with detectable plasma DA levels. This is consistent with the fact that DA is synthesized extraneuronally in proximal tubular cells and that DA excreted into urine is almost exclusively formed within the kidney (Lee 1993; Carey 2001). The intrarenal dopaminergic system also modifies the renal capacity of natriuresis. For instance, under salt deprivation, Ang II has a central role in t_Na_ (Crowley and Coffman 2007), whereas under a high‐salt diet, DA secreted by renal proximal tubules acts as a paracrine substance to inhibit t_Na_ along the proximal to distal nephron (Bertorello et al. 1988; Siragy et al. 1989; Bertorello and Aperia 1990; Olsen 1998; Carey 2001; Féraille and Doucet 2001; Gildea et al. 2012). Intrarenally produced DA inhibits Na^+^‐K^+^‐ATPase (Aperia et al. 1987; Carey 2001) and Na^+^/H^+^ exchanger activity (Felder et al. 1990) of tubular cells to increase urinary sodium excretion via both D1‐like and D2‐like receptors. In patients of the present study, whose daytime U_Na_V increased, an increase in U_DA_V was accompanied by an increase in filtered tubular Na load, consistent with the finding that as the amount of sodium delivered to proximal tubules increases, DA secreted by the tubules is enhanced. At baseline, U_K_V/U_Na_V ratio did not relate to U_DA_V, and change in U_K_V/U_Na_V correlated inversely with change in U_DA_V. These findings are also concordant with clinical findings that, on a low‐salt diet, the intrarenal dopaminergic system cannot exert sufficient natriuresis, but does decrease renal distal tubule sodium transport with RAAS inhibition (Seri et al. 1990; Natarajan et al. 2016), since our study population were on a relatively low‐salt diet. The interaction between the intrarenal RAAS and dopaminergic system can be altered by dietary salt intake, and both systems play an important pathophysiological role in development of salt‐sensitive hypertension, and thus, nondipper circadian BP rhythm. In our study, only five patients had dipper type circadian BP rhythm. Therefore, we could not compare the results by dividing subjects into dippers and nondippers. Fundamental limitation of our study is that we measured too many variables despite a small number of patients. Also, the number of males and females differ, and the three treatment doses of ARB were adopted. Another limitation of our study is lack of investigating the expression levels of AngII type 1 and type 2 receptors (AT1R and AT2R), and D1‐like and D2‐like receptors. DA receptors decrease AT1R and can cooperate with AT2R to increase tubular sodium reabsorption, and D1‐like receptors also reduce the effect of AT2R inhibition caused by AngII (Gildea et al. 2008, 2012). A reduced level of D1‐like receptors can also contribute to salt‐sensitive hypertension (Luippold et al. 2001).

### Heart rate variability

The sympathetic nerve system can stimulate t_Na_, whereas ARBs inhibit central and peripheral sympathetic nerve activity (Ye et al. 2002). Recently, we proposed an increase in the non‐Gaussianity index of HRV, *λ*_25s_, which indicates the probability of volcanic heart rate deviations of departure from each standard deviation level, as a marker of sympathetic cardiac overdrive (Kiyono et al. 2008; Hayano et al. 2011). We also reported that the L/T‐type calcium channel blocker, azelnidipine, which has been shown to decrease sympathetic nerve activity in experimental (Shokoji et al. 2005; Konno et al. 2008) and clinical studies (Inomata et al. 2014), reduces *λ*_25s_ in CKD patients under preceding treatment with ARBs (Fukuda et al. 2016). Therefore, the present study was performed to evaluate the relationship between HRV and urinary sodium excretion or circadian BP rhythm. At baseline, *λ*_25s_ was higher and DC was lower compared to control values. However, HRV did not change during the acute phase of ARB treatment, and there were no significant relationships among HRV, Na dynamics, and the intrarenal RAAS and dopaminergic system. We speculate that these results reflect the balance between the sympathoinhibitory effect of the ARB and sympathetic reflex in response to BP reduction.

We have also proposed that *λ*_25s_ is closely related to sympathetic nervous activity, HF is related to the parasympathetic nervous system, and DC to both the sympathetic and parasympathetic nervous systems (Fukuda et al. 2016). The present and previous studies consistently showed that GFR had a positive correlation with DC, but not with HF or *λ*_25s_ (Fukuda et al. 2016). A further study is needed to investigate the difference in the relationships of these HRVs with GFR.

The present study did suggest a solution to the issue of HRV in relation to circadian BP rhythm. Night/day BP ratio and nocturnal BP were attributable to HF, rather than DC or *λ*_25s_, indicating that nocturnal hypertension or nondipper circadian BP rhythm can be due to lost parasympathetic nervous activity, rather than activated sympathetic nervous system, at night. Since investigation of receptor function (i.e., AngII and dopaminergic system) is saddled with the issue of sensitization and desensitization, we performed this study during the acute phase of ARB treatment, rather than the chronic phase. A further study is needed to investigate whether the systemic sympathetic nervous system and intrarenal dopaminergic system are both significant for renal sodium handling, or whether the dopaminergic system rather than the sympathetic nerve system is significant, in the chronic phase of ARB treatment.

## Conclusions

In conclusion, as renal function deteriorated, diminished sodium excretion caused the nondipper type of circadian BP rhythm, which can be attributed to intrarenal RAAS and dopaminergic system and injured parasympathetic nerve activity. During the acute phase of treatment, the sympathoinhibitory effect of ARBs cannot contribute to an increase in natriuresis or decrease in nocturnal BP. Instead, ARBs inhibit intrarenal RAAS to enhance urinary sodium excretion and restore circadian BP rhythm in cooperation with the intrarenal dopaminergic system.

## Conflict of Interest

None declared.
